# Pregnancy-Associated Spontaneous Coronary Artery Dissection in Women: A Literature Review

**DOI:** 10.1016/j.curtheres.2023.100697

**Published:** 2023-03-01

**Authors:** Katherine Zeven

**Affiliations:** Warren Alpert Medical School, Brown University, Providence, Rhode Island

**Keywords:** postpartum and pregnancy, SCAD, spontaneous coronary artery dissection, spontaneous coronary artery dissection and pregnancy

## Abstract

**Background:**

Spontaneous coronary artery dissection (SCAD) primarily affects women younger than age 50 years, is often misdiagnosed or undiagnosed, and research on this topic is limited.

**Objective:**

A literature review was conducted to identify unique factors that can facilitate pregnancy-related SCAD (P-SCAD) diagnosis as well as differentiate it from nonpregnancy-related SCAD (NP-SCAD).

**Methods:**

A literature search was conducted on PubMed, Medline, Embase, The Cochrane Database of Systematic Reviews, and Google Scholar databases that focused on NP-SCAD and P-SCAD cases reported in North America between 2006 and 2021 using the terms *spontaneous coronary artery dissection, misdiagnosis*, and *women*, along with *postpartum* and *pregnancy*. The Let Evidence Guide Every New Decision quality assessment tool was applied to all reviews.

**Results:**

A total of 108 journal articles reporting on individual cases, case series examining independent SCAD registries, as well as literature reviews were identified. These included 1547 SCAD cases in women, 510 of which were identified as P-SCAD. SCAD occurs predominantly in women, and thus presents a diagnostic challenge because women are not typically considered at risk for developing cardiovascular diseases and may present with symptoms that mimic other medical conditions. This issue is further exacerbated when SCAD develops during pregnancy or the postpartum period (ie, P-SCAD to differentiate it from SCAD occurring in other periods of woman's life such as NP-SCAD) because P-SCAD patients often present with less typical cardiac symptoms yet tend to experience more severe illness that can jeopardize their health and that of their baby. P-SCAD was associated with higher ST-segment elevation myocardial infarction rates, higher troponin levels, and a greater risk of cardiogenic shock compared with NP-SCAD cohorts. It was also evident that the failure rates associated with invasive procedures such as percutaneous coronary intervention and coronary artery bypass graft surgery were higher in P-SCAD patients, whereas the mortality rates are comparable to NP-SCAD cohorts if diagnosed and treated appropriately.

**Conclusions:**

Because younger women are rarely screened, they are at greater risk from SCAD, especially if this condition develops during pregnancy or ≤30 days following delivery. It is essential that medical professionals providing care for pregnant women understand P-SCAD risk factors and provide medical counseling for pregnant women or those planning a pregnancy to be better equipped to recognize its more subtle signs and symptoms, thus facilitating timely specialist referral, diagnosis, and treatment. (*Curr Ther Res Clin Exp*. 2023; 84:XXX–XXX)

## Introduction

Spontaneous coronary artery dissection (SCAD) is defined as “an acute manifestation of a false, separate lumen within the coronary artery, either caused by an internal tear or an acute bleeding within the tunica media of the arterial wall not resulting from trauma or atrogenic separation.”^1^ At present, 3 SCAD types are formally recognized, and a fourth type is suggested.^2^^,^^3^ The main consequences of SCAD are acute myocardial infarction (MI) and cardiogenic shock. Because of the presence of relatively few risk factors, especially in young women, prompt diagnosis is critical to appropriate patient management.

Whereas SCAD primarily affects women (who account for 87%−95% of all known cases)^4^ and more frequently presents in younger women (aged <50 years),^5^ it is routinely misdiagnosed if no cardiac risk factors are present because women are typically not considered at high risk for developing cardiovascular issues.^6^ Given that pregnancy exacerbates the SCAD risk as well as its severity,^7^ its timely diagnosis and treatment are critical.^8^, ^9^, ^10^ For this purpose, it is important to distinguish between pregnancy-related SCAD (ie, SCAD that develops during pregnancy and/or the first 12 weeks postpartum [P-SCAD]) and that which occurs in any other period of woman's life (ie, nonpregnancy-related SCAD [NP-SCAD]).

This literature review was conducted to identify the main factors that may assist with P-SCAD diagnosis as well as its differentiation from NP-SCAD. Its further aim is to assess the effectiveness of different treatments and the likelihood of SCAD recurrence after pregnancy.

## Methods

### Population

#### Search strategy

The literature included in this review was obtained by searching PubMed, Medline, Embase, The Cochrane Database of Systematic Reviews, and Google Scholar online databases using the terms *spontaneous coronary artery dissection, spontaneous coronary artery dissection and pregnancy, misdiagnosis*, and *women* along with *postpartum* and *pregnancy*.

#### Inclusion and exclusion criteria

Precedence was given to articles describing NP-SCAD and/or P-SCAD cases reported in North America between 2006 and 2021, literature reviews, and case series examining independent SCAD registries. P-SCAD was based on SCAD occurring during pregnancy, up to 12 weeks thereafter, unless a longer period was otherwise noted. Portions of reviews which contained male and female patients with SCAD were excluded if symptoms and treatment data was not differentiated between men and women. Individual case series included in literature reviews were excluded to avoid double counting of results, as were restatements of prior studies that contained duplicative results.

#### Identified sources

As a result of this strategy, 108 articles, literature reviews, and case series referencing the search terms were reviewed.

#### Statistics

Descriptive statistics were summarized as mean (SD), median (interquartile range), or counts and percentages.

#### Method for collating data

For the SCAD cases identified, data were sorted by women and further sorted by P-SCAD cases, which were reviewed to establish the main symptoms, treatments, and outcomes. The results shown reflect the data identified in the review only and may not include all cases of P-SCAD occurring during the period. The author reviewed, collected, and collated the data.

### Data

For all identified cases, the patient's age, family history, and traditional cardiac risk factors (eg, hypertension, obesity, and smoking), as well as history of hyperlipidemia were gathered, along with pregnancy-related data if applicable/available, including preeclampsia, use of oral contraceptives, hormone therapy, time of SCAD occurrence during and after pregnancy, prior pregnancies, and SCAD recurrence. Timing of pregnancy, miscarriage, or postpartum status, and associated pathologies such as fibromuscular dysplasia (FMD), systemic inflammatory disease, and connective tissue disorder, as well as presence of chest pain, troponin levels, and emotional and physical stressors, were also recorded if reported by the original investigators.

## Results

A total of 108 articles were identified from all search engines, of which 6 were duplicates. After exclusion of 48 articles and 6 duplicates, 54 articles were reviewed, including 13 case series and 6 case reports. This totalled 1547 female SCAD patients, of whom 510 were P-SCAD patients.

Table 1 contains the main articles used for the majority of the findings contained herein, along with the sample characteristics and analysis performed.

**Table 1:** Main articles, case studies reviewed, sample characteristics, and analysis performed.*

	No. of P-SCAD cases	No. of NP-SCAD cases	Timing of SCAD during pregnancy	Diagnostic methods including angiography, OCT, and IVUS	STEMI	Presence of FMD or other immune disorders	Conservative treatment method
Saw et al^11^	54	610	34 (5)	548 (83)	223 (34)	233 (35)	573 (86)
Havakuk et al^12^	120	0	119 (99)	0 (0)	90 (75)	0 (0)	54 (45)
Tweet et al^13^	54	269	54 (15)	0 (0)	30 (9.2)	26 (8)	22 (7)
Tweet et al^14^	23	69	23 (25)	0 (0)	8 (8.7)	0 (0)	43 (47)
Faden et al^15^	79	0	0 (0)	31 (39)	42 (63.6)	0 (0)	27 (34)
Elkayam et al^16^	150	0	150 (100)	129 (86)	105 (70)	0 (0)	129 (86)
Cade et al^17^	13	0	13 (100)	0 (0)	6 (46)	0 (0)	7 (54)
Numasawa et al^18^	1	0	0 (0)	1 (100)	1 (100)	0 (0)	0 (0)
Manasrah et al^19^	3	0	3 (100)	3 (100)	0 (0)	0 (0)	0 (0)
Yogeswaran et al^20^	1	0	1 (100)	1 (100)	1 (100)	0 (0)	0 (0)
Lee et al^21^	1	0	1 (100)	0 (0)	1 (100)	1 (100)	0 (0)
Sharma et al^22^	9	89	98 (100)	0 (0)	0 (0)	0 (0)	0 (0)
Davis et al^23^	1	0	1 (100)	0 (0)	0 (0)	0 (0)	1 (100)
Elshatanoufy et al^24^	1	0	1 (100)	1 (100)	1 (100)	0 (0)	0 (0)

FMD = fibromuscular dysplasia; IVUS = intravascular ultrasound; NP-SCAD = nonpregnancy-related spontaneous coronary artery dissection; OCT = optical coherence tomography; P-SCAD = pregnancy-related spontaneous coronary artery dissection; STEMI = ST-segment elevation myocardial infarction.

⁎ Values are presented as n (%).

### Population characteristics

Before commencing the review, the data pertaining to 1547 female SCAD patients identified in extant literature was examined to segregate P-SCAD patients (n = 510) from NP-SCAD patients (n = 1037) to compare and contrast the findings with the aim of aiding timely diagnosis and treatment. With the exception of 1 article reporting on a prospective study^11^ the remaining publications were based on retrospective studies involving data retrieved from SCAD databases and provided by treating physicians or single case studies. As expected, given the limitations of reproductive age, the P-SCAD cohort was younger (aged 17−52 years, mean age = 27.9 years) than SCAD patients (aged 24−89 years, mean age = 43.7 years) and generally presented with fewer risk factors for SCAD than NP-SCAD patients. On the other hand, in both cases, women tended to report similar symptoms. Initial presentation and symptoms were reported to include chest pain, back pain, headache, shortness of breath, and dizziness for both P-SCAD and NP-SCAD patients.

### Predisposing conditions and associated pathologies

As can be seen from Table 2 contrasting pathology related to P-SCAD and NP-SCAD cases, the former are much more difficult to identify, owing to a small number of risk factors that would prompt screening for SCAD. The most frequent associated pathology reported was FMD. The incidence rates of FMD for P-SCAD and NP-SCAD cases are divergent, with NP-SCAD cases experiencing twice the rate of FMD occurrence, when viewing all screens (partial and complete) for FMD. NP-SCAD cases included associated pathologies of systemic inflammatory disease were more than double the P-SCAD cases (see Table 2). Connective tissue disorder occurred twice as much in P-SCAD cases when compared with NP-SCAD cases, although the data sample was relatively small.^11^ For this reason, as noted by Saw et al^11^ a majority of women are not screened for FMD, or are provided with incomplete FMD screening, which may contribute to misdiagnosis of P-SCAD.

**Table 2:** Associated pathologies present in pregnancy-related spontaneous coronary artery dissection (P-SCAD) vs nonpregnancy related SCAD (NP-SCAD).*

	P-SCAD cases	NP-SCAD cases
Fibromuscular dysplasia (complete screens)	(n = 63) 17 (27)	(n = 411) 233 (56.7)
Fibromuscular dysplasia (including all screens)	(n = 63) 17 (27)	(n = 750) 550 (73.3)
Systemic inflammatory condition	(n = 54) 1 (1.8)	(n = 664) 35 (5.3)
Connective tissue disorder	(n = 23) 2 (8.7)	(n = 664) 27 (3.6)

⁎ Values are presented as n (%). Sources: Saw et al,^11^ Havakuk et al,^12^ Tweet et al,^13^ Tweet et al,^14^ Faden et al,^15^ Elkayam et al,^16^ Cade et al,^17^ Numasawa et al,^18^ Manasrah et al,^19^ Yogeswaran et al,^20^ Lee et al,^21^ Sharma et al,^22^ Davis et al,^23^ and Elshatanoufy et al.^24^

### Presentation

#### P-SCAD timing during pregnancy

The findings pertaining to SCAD onset are reported in Table 3^11^, ^12^, ^13^, ^14^, ^15^, ^16^, ^17^, ^18^, ^19^, ^20^, ^21^, ^22^, ^23^, ^24^ in P-SCAD patients (n = 510). The largest occurrence of P-SCAD occurred during pregnancy or within 30 days after delivery. The third trimester saw the higher rate of P-SCAD during pregnancy. However, the greatest occurrence of P-SCAD occurred during the peripartum period, defined as immediately before or within 1 week after birth, and the postpartum period up to ≤30 days, comprising 37.6% of all occurrences in the P-SCAD patients reviewed.^6^ Data collection is not always consistently reported for the postpartum period, oftentimes referred to as occurring ≤30 days, ≤12 weeks, or ≤12 months postpartum.^8^^,^^11^^,^^13^^,^^14^^,^^17^

**Table 3:** Timing of pregnancy-related spontaneous coronary artery dissection (P-SCAD) diagnosis during pregnancy.*

	Saw et al^11^	Havakuk et al^12^	Tweet et al^13^	Tweet et al^14^	Faden et al^15^	Elkayam et al^16^	Cade et al^17^	Sharma et al^22^	Other^18^, ^19^, ^20^, ^21^, ^22^, ^23^, ^24^
No. of P-SCAD cases	n = 54	n = 120	n = 54	n = 23	n = 79	n = 150	n = 13	n = 9	n = 8
Unknown period during pregnancy	1 (1.8)	1 (1)	4 (7.4)	2 (8.7)	0 (0)	11 (7)	0	9 (100)	1 (13)
First trimester	0 (0)	0 (0)	1 (2)	0 (0)	0 (0)	9 (6)	0	0 (0)	1 (13)
Second trimester	0 (0)	7 (6)	0 (0)	0 (0)	0 (0)	17 (11)	0	0 (0)	0 (0)
Third trimester	0 (0)	21 (17.5)	1 (2)	0 (0)	20 (25)	40 (27)	1 (8)	0 (0)	1 (13)
Peripartum	34 (62.9)	4 (3)	0 (0)	0 (0)	4 (5)	66 (44)	0	0 (0)	0 (0)
≤30 d after delivery	0 (0)	0 (0)	19 (35)	1 (5)	55 (69.6)	7 (5)	12 (92)	0 (0)	4 (50)
≤12 wk after delivery or breastfeeding	19 (35.2)	87 (72)	29 (54)	7 (30)	0 (0)	0 (0)	0	0 (0)	1 (13)

⁎ Values are presented as n (%). Sources: Saw et al,^11^ Havakuk et al,^12^ Tweet et al,^13^ Tweet et al,^14^ Faden et al,^15^ Elkayam et al,^16^ Cade et al,^17^ Numasawa et al,^18^ Manasrah et al,^19^ Yogeswaran et al,^20^ Lee et al,^21^ Sharma et al,^22^ Davis et al,^23^ and Elshatanoufy et al.^24^

#### Clinical findings

As can be seen from Table 4, P-SCAD and NP-SCAD cases can also be distinguished in terms of ST-segment elevation MI presence and location, as well as very high troponin levels, whereas the frequencies of moderately elevated troponin levels are comparable.

**Table 4:** Troponin levels and ST-segment elevation myocardial infarction in pregnancy-related spontaneous coronary artery dissection (P-SCAD) vs nonpregnancy-related SCAD (NP-SCAD).

	P-SCAD cases	NP-SCAD cases
Elevated troponin levels†	(n = 40) 38 (95)	(n = 716) 699 (97.6)
Very high troponin levels‡	(n = 40) 11 (28)	(n = 716) 83 (11.6)
ST-segment elevation myocardial infarction	(n = 367) 236 (64)	(n = 98) 49 (50.0)

† Elevated troponin levels are a marker of myocardial injury and are found in virtually all presentations ultimately diagnosed with SCAD. Anything above the normal range (0 and 0.4 ng/mL) is considered to be an elevated troponin level in the blood.

‡ Very high troponin levels occur as >500 × upper limit of normal.

#### Precipitating stressors

When P-SCAD and NP-SCAD cases were compared with respect to known stress factors that can precipitate SCAD (Table 5), it was apparent that these were much more prevalent in the latter cohort, making P-SCAD cases even harder to predict.

**Table 5:** Precipitating stressors/risk factors pregnancy-related spontaneous coronary artery dissection (P-SCAD) vs nonpregnancy-related SCAD (NP-SCAD).*

	P-SCAD cases	NP-SCAD cases
Emotional stress	(n = 91) 15 (16.4)	(n = 762) 428 (56.2)
Physical stress	(n = 91) 2 (2.2)	(n = 762) 238 (31.2)
Heavy lifting	(n = 68) 0 (0)	(n = 664) 74 (11.1)
Valsalva type maneuvers	(n = 34) 0 (0)	(n = 664) 90 (13.6)

⁎ Values are presented as n (%). Sources: Saw et al,^11^ Havakuk et al,^12^ Tweet et al,^13^ Tweet et al,^14^ Faden et al,^15^ Elkayam et al,^16^ Cade et al,^17^ Numasawa et al,^18^ Manasrah et al,^19^ Yogeswaran et al,^20^ Lee et al,^21^ and Sharma et al.^22^

### Diagnosis

There was no specific alternative diagnosis strategy described in P-SCAD. No meaningful separate data on P-SCAD diagnostic methods was reported. Diagnostic methods used to identify SCAD were reported as follows: angiography on initial diagnosis (n = 670) was reported in 495 cases (73.9%), optical coherence tomography (n = 670) in 41 cases (6.1%), and intravascular ultrasound (n = 670) in 18 cases (2.7%).^11^, ^12^, ^13^, ^14^, ^15^, ^16^, ^17^, ^18^, ^19^, ^20^, ^21^, ^22^ Rates of use for these methods followed conventional diagnostic practice.

### Treatment approaches

#### General

As can be seen from Table 6, conservative approach is more likely to be adopted for NP-SCAD relative to P-SCAD patients, who are more likely to progress to revascularization and require percutaneous coronary intervention (PCI), which is frequently unsuccessful. Amongst P-SCAD patients, treatment strategy was disparate amongst different authors. When pooled, an initial conservative approach was used in 42.7% of P-SCAD cases; however, this varied from 34.1% to 53.8%. An initial PCI was performed in 41.6% of P-SCAD patients at admission, whereas only 20.8% of SCAD patients reported an initial PCI at admission. Of the patients receiving initial conservative treatment, 32.2% of P-SCAD patients had to be referred to revascularization, whereas only 2.3% of SCAD patients needed further invasive treatment. Of the patients initially treated with PCI, the 47 P-SCAD cases experienced PCI failure (32.9%), whereas 25 NP-SCAD cases experienced PCI failure (20.3%). When comparing P-SCAD with NP-SCAD patients, NP-SCAD patients were more likely to be treated using a conservative approach (77.2% vs 42.7%).

**Table 6:** Aggregate initial treatment of P-SCAD vs. NP-SCAD cases

	P-SCAD cases n (%)	NP-SCAD cases n (%)
	n = 363	n = 1,019
Conservative Treatment	155 (42.7)	787 (77.2)
Progressed to Revascularization	39 (32.2)	18 (2.3)
Percutaneous coronary intervention (PCI)	151 (41.6)	212 (20.8)
Unsuccessful PCI	47 (32.9)	25 (20.3)

Sources: Saw et al,^11^ Havakuk et al,^12^ Tweet et al,^13^ Tweet et al,^14^ Faden et al,^15^ Elkayam et al,^16^ Cade et al,^17^ Numasawa et al,^18^ Manasrah et al,^19^ Yogeswaran et al,^20^ Lee et al,^21^ and Sharma et al.^22^

#### Conservative approach

Treatment involving conservative approach also differs considerably depending on whether SCAD occurs during pregnancy or postpartum, or at another time in a woman's life, as indicated in Tweet et al's^13^ 2017 detailed review of P-SCAD cases (n = 54) and NP-SCAD cases (n = 269) in Table 7. Once again, the most significant difference relates to the number of P-SCAD patients who progress to revascularization, which is significantly higher than that pertaining to NP-SCAD. These patients are also more likely to require coronary artery bypass graft (CABG) procedure and experience adverse consequences due to unsuccessful treatment, including maternal death.^13^

**Table 7:** 2017 Tweet et al^13^ initial treatment P-SCAD v. NP-SCAD cases (n = 323)

	P-SCAD cases n (%)	NP-SCAD cases n (%)
	n = 54	n = 269
Conservative Treatment	22 (40.7)	139 (52)
Progressed to Revascularization	3 (13.6)	3 (1.6)
PCI	23 (42.6)	123 (45.7)
Unsuccessful PCI	8 (35)	25 (20)
Coronary Artery Bypass Graft CABG)	14 (26)	19 (7)

Source: Tweet et al^13^

#### Revascularization

Expanding the 2017 study in 2020, Tweet et al^14^ reported similar results for initial conservative management in P-SCAD, reflecting 46.7% of P-SCAD patients experiencing initial conservative management, with 8% referred to PCI.

### Recurrence

Given the potentially devastating consequences of SCAD, especially during pregnancy, its recurrence was also investigated,^6^^,^^12^, ^13^, ^14^^,^^21^, ^22^, ^23^, ^24^, ^25^, ^26^, ^27^ and the findings are reported in Table 8. Although very few authors reported these data based on a limited number of cases, including SCAD patients from the Mayo Clinic SCAD registry with child-bearing potential, it appears that the risk is relatively low, but does increase after 2 years following delivery in women who have previously had SCAD or P-SCAD.

**Table 8:** Recurrence of spontaneous coronary artery dissection after pregnancy in prior spontaneous coronary artery dissection/pregnancy-related spontaneous coronary artery dissection patients.*

Postpartum	Tweet et al^13^ (n = 54)	Tweet et al^14^ (n = 23)	Davis et al^23^ and Elshatanoufy et al^24^ (n = 2)
≤12 wk after delivery	4 (7.4)	0 (0)	0 (0)
>12 wk after delivery	0 (0)	0 (0)	0 (0)
>13 mo after delivery	0 (0)	0 (0)	0 (0)
>2 y after delivery	4 (7.4)	2 (8.7)	2 (100)

⁎ Values are presented as n (%). Sources: Saw et al,^11^ Hayes et al,^6^ Tweet et al,^13^ Tweet et al,^14^ Lee et al,^21^ Sharma et al,^22^ Davis et al,^23^ Elshatanoufy et al,^24^ Clare et al,^25^ Tweet Eleid et al,^26^ and Saw et al.^27^.

Beyond the Tweet et al^13^^,^^14^ studies, 2 other cases relevant to the study of SCAD recurrence and pregnancy were reported by Davis et al^23^ and Elshatanoufy et al.^24^ Davis et al^23^ reported 1 case with no SCAD recurrence during a 3-year follow-up after pregnancy in a 34-year-old patient after a P-SCAD incident 2 weeks following a first trimester miscarriage. Elshatanoufy et al^24^ reported no recurrence in a SCAD patient who had a prior P-SCAD occurrence 9 weeks postpartum.^24^

## Discussion

### Presentation

Because SCAD affects young women not otherwise presenting with traditional cardiovascular disease risk factors, it has recently gained considerable attention among researchers and practitioners. Available evidence indicates that women account for 87% to 95% of all recorded SCAD cases,^4^ and 35% of MI in young women are ascribed to SCAD.^20^^,^^28^^,^^29^ The affected patients typically present with chest pain (describing it as crushing pressure or sharp pain), but may also report back pain, hypertension, and/or shortness of breath. Because these symptoms are also indicative of (obstructive) atherosclerotic acute coronary syndrome (ACS), there is a risk of emergency department misdiagnosis or delayed SCAD diagnosis.^1^^,^^31^ This issue is further exacerbated by the fact that in 33% of SCAD patients, electrocardiograph tests^7^ yield normal findings and vital signs are normal.^31^ According to Lindor et al,^31^ hypertension is most prevalent in SCAD cases, whereas Giacoppo et al^32^ caution that absence of such symptoms does not rule out SCAD. These inconsistencies, along with a limited number of presenting symptoms, make it difficult to arrive at an accurate diagnosis. This issue is exemplified by the fact that women younger than age 50 years experiencing ACS symptoms are 7 times more likely to be misdiagnosed and discharged by hospital emergency department staff,^5^ possibly due to the perception that women, especially young women, are not readily susceptible to cardiac issues. If other symptoms not usually associated with cardiac disease—such as back pain, shortness of breath, fatigue, headache, and dizziness—are also present, the potential for misdiagnosis will increase further.^5^

### Risk factors

SCAD during pregnancy can pose a significant risk, putting about 1.8% of every 100,000 pregnant women in the United States in danger.^10^^,^^15^ As pointed out by Tweet et al^13^ and Havakuk et al,^12^ very few P-SCAD cases are reported in the literature, and those described involve even fewer traditional cardiac risk factors than NP-SCAD cases. Because P-SCAD patients tend to primarily complain of nonspecific chest and back pain, P-SCAD is challenging to diagnose.^11^, ^12^, ^13^, ^14^, ^15^, ^16^, ^17^, ^18^, ^19^, ^20^, ^21^, ^22^

To assist with this process, Sharma et al^22^ and Saw et al^11^ provided a comprehensive listing of risk factors—including those traditionally associated with ACS, such as hypertension (occurring in 27% or 32.1% cases), hyperlipidemia (14%), dyslipidemia (20.3%^8^), and smoking (22% or 11.6%)—but placing emphasis on gestational hypertension (14%), preeclampsia (8%), and gestational diabetes, as well as prior fertility treatment (15%) and hormone replacement therapy (47% of the postmenopausal women).^20^

These assertions are supported by the findings yielded by a number of SCAD/pregnancy-focused studies, including Saw et al's^3^ prospective multicenter SCAD review across Canada, where P-SCAD patients experiencing acute MI were found to be more likely to have fewer risk factors than NP-SCAD patients.^12^^,^^13^^,^^17^^,^^20^^,^^33^ Because these results pertain to mostly Caucasian women, it is worth noting that they are corroborated by the findings yielded by Clare et al's^25^ more ethnically diverse study. Thus, although race does not appear to influence the identification of P-SCAD, accurate representation of diverse populations in SCAD registries is warranted.

Available evidence further indicates that pregnancy-related physiological changes present risk factors for SCAD.^34^ Indeed, although P-SCAD accounts for <10% of SCAD diagnoses in women, SCAD is associated with up to 43% of acute MI cases that occur during pregnancy, as well as 50% of the ACS cases diagnosed during the postpartum period.^35^^,^^36^ These findings are attributed by Maas et al^35^ to the high progesterone levels and the rapid changes in hormones at birth and during the postpartum period.^35^ The authors challenge the prevalent view that hormones (such as contraceptives and hormone replacement therapy) and changes in hormone levels during birth are risk factors for SCAD because SCAD incidence is low compared with the widespread application of those hormones. Likewise*,* Saw et al^37^ and Alterie et al^33^ posit that other stressors and arteriopathies can contribute to the emergence of SCAD, given that psychological and physical precipitating stressors have been identified as risk factors.^11^

#### Troponin

As was demonstrated earlier, troponin is elevated in all women who are ultimately diagnosed with SCAD, but much higher levels are noted in P-SCAD patients.^11^ Troponin is an enzyme generated either when the heart muscle is damaged or by other muscles during heavy, intense exercise. Whereas elevated troponin levels are a marker of myocardial injury and are found in virtually all presentations ultimately diagnosed with SCAD, P-SCAD patients appear to be more severe (more proximal lesions, multivessel lesions, and hemodynamic instability), which may explain the reported instances of very high levels of troponin in P-SCAD patients.

#### Non-ST-elevation MI and ST-elevation MI

P-SCAD patients are also more likely to present with ST-elevation MI than non-ST-elevation MI patients and their ST values tend to be higher than in NP-SCAD cases.^13^ Because greater prevalence of left main dissections as well as vertebral dissections results in more extensive injury,^12^^,^^13^^,^^15^^,^^20^^,^^33^ which can lead to vascular spasms, P-SCAD patients require careful observation and timely treatment.^1^^,^^38^

#### Timing of P-SCAD during pregnancy

Because P-SCAD most frequently occurs during the last semester and the first week following delivery,^28^ women deemed at risk should be given extensive care during this period and should be monitored by both cardiologist and obstetrician, as noted by Yip et al.^8^ However, given that in many cases P-SCAD may not cause any complaints, as noted by Alterie et al,^33^ identifying patients who require this level of care may be challenging and would require close examination of patient history.^15^ Given that P-SCAD has been shown to be predominantly present in women of various childbearing ages, Tweet et al's^14^ uniquely focused 2020 study of the relationship between SCAD recurrence and pregnancy should be expanded. The potential relationship between other factors such as emotional and physical stress, in vitro fertilization, progesterone levels, fertility treatments, birth control, hormone therapy, menses-related chest pain, and oral contraception use should be examined to further identify risk factors and causes associated with P-SCAD.^11^

In the 2017 Mayo Clinic SCAD registry study, Tweet et al^13^ reported that the highest incidents of P-SCAD occurred postpartum. Although there is general agreement that the majority of P-SCAD occurs postpartum, data collection is not always consistently reported (eg, peripartum, postpartum ≤30 days, postpartum ≤12 weeks or ≤12 months).^8^^,^^11^^,^^13^^,^^14^^,^^17^ Although there is divergence in data collection due to lack of uniformity, it is acknowledged that the majority of P-SCAD incidents occur postpartum within 30 days and often mostly within the first week after giving birth.^8^^,^^28^ Faden et al^15^ support this, noting that when compared with a general population exhibiting ACS of 3% to 4%, the incidence of P-SCAD may be underreported.^15^

### Predisposing conditions and associated pathologies

#### FMD

Associated pathologies such as FMD, systemic inflammatory disease, and connective tissue disorder are commonly present in SCAD patients,^13^ with incidence rates ranging from 40% to 86%.^1^^,^^22^^,^^26^^,^^27^^,^^39^ Similar to SCAD, FMD affects younger women and is also presently underdiagnosed.^39^^,^^41^ Although the FMD−SCAD association was first reported by Saw et al^39^ in 2012, limited data exist on the prevalence of noncoronary FMD among SCAD patients. However, recent reports purporting a genetic link between SCAD and FMD suggest that both conditions are noninflammatory in nature and are not a result of an autoimmune disease.^39^ Kronzer et al^42^ note that the association between FMD and SCAD is statistically significant when compared with the general population. A recently noted genetic link between SCAD and FMD may explain the association.^42^

Although the available data point to a significant variance in the presence of FMD between P-SCAD and NP-SCAD cases,^4^^,^^11^^,^^37^^,^^43^ this discrepancy is likely due to small sample sizes and inconsistency in screening protocols adopted for FMD. Data for FMD where only complete FMD screening was performed showed a 56.7% presence of FMD of the population fully screened. Given that only 54.8% of that cohort was tested for FMD, and FMD was shown in 56.7% of those patients, the prevalence of FMD is underrepresented due to the incomplete screening.^11^^,^^39^ Notwithstanding any differences found among P-SCAD and NP-SCAD cases in the reported studies, the elevated presence of FMD is still significant enough to be a valuable tool for evidencing SCAD in both populations.

Because FMD affects the artery walls, causing them to lose flexibility and become weak,^30^^,^^39^ its greater occurrence in women relative to men implicates estrogen—along with other hormonal factors relating to fertility treatments, chemical contraception, hormone replacement therapy, and pregnancy—in its emergence.^4^ Whereas the etiology of FMD is still not known, both Saw et al^11^ and Tweet et al^40^ suggest such a high correlation between FMD and SCAD reveals a causal link, not merely an association. Kronzer et al^42^ suggest a susceptibility to SCAD may be genetic, given the potential genetic link between FMD and SCAD.^42^ It is not fully understood if these conditions are underlying causes or occur simultaneously with SCAD. Some research indicates that it is a predisposing condition.^1^^,^^4^^,^^11^ The high correlation with SCAD should be an indicator that once FMD has been established, a SCAD diagnosis is likely.^41^ Further study of the prevalence of FMD in P-SCAD patients could provide insight into a link to SCAD and P-SCAD

#### Systemic inflammatory disease and connective tissue disorder

Although some SCAD patients have inheritable connective tissue disorders such as Marian syndrome, Ehlers-Danlos, and Loeys-Dietz,^42^ their prevalence in P-SCAD patients is estimated to be below 2.5%.^9^ However, because a limited number of women are offered screening for systemic inflammatory disease and connective tissue disorder, it is difficult to establish the prevalence rates in P-SCAD and NP-SCAD cohorts or compare the findings.

In a 2019 study, Saw et al^11^ reported systemic inflammatory disease and connective tissue disorder found in 5.27% and 3.6% of SCAD patients, respectively. Because most of the SCAD patients in the study were not screened at all for systemic inflammatory disease or connective tissue disorder, those rates could be higher, consistent with FMD. In a prior 2014 study, Saw et al^44^ reported that prevalence for systemic inflammatory disease in SCAD patients was higher at 8.9%.^44^ The later study's lower rate of 5.27% suggests that further screening and study is warranted. Although the reports show a correlation, often high, no link between systemic inflammatory disease and SCAD has been established.^1^ Saw et al^43^ suggest such a link and later observed^11^ that systemic inflammatory disease and connective tissue disorder are predisposing. However, no significant difference between P-SCAD and SCAD cases has been established for the presence of these 2 disorders.

### Precipitating stressors

Because pregnancy, emotional stress, and physical stress are known stressors for SCAD, they are expected to exacerbate P-SCAD. Extant findings counter this assumption because they are mostly based on self-reports by women and their reliability is questionable.^13^^,^^14^^,^^22^

### Diagnosis

Given that SCAD is a multifactorial disease, several diagnostic methods and data evaluation strategies should be utilized to ensure accurate diagnosis. Angiography is considered the gold standard^10^^,^^11^^,^^45^ for diagnosing SCAD, especially in the absence of recognized coronary risk factors. Additional intravascular imaging, such as intravascular ultrasound or optical coherence tomography, may be required for confirmation when the most common form of SCAD, type 2, is suspected, because its structure may preclude accurate diagnosis.^3^^,^^10^^,^^17^ Moreover, given that there are no blood markers for SCAD or P-SCAD, troponin levels should be checked because troponin values are typically elevated in the blood of these patients.

Because high progesterone levels during pregnancy and hormonal changes immediately upon delivery have been shown to contribute to P-SCAD emergence, additional care is required when treating women that have a history of multiple births and preeclampsia.^35^

More research is needed to discover potential triggers during pregnancy, which is also supported by Tweet et al^13^^,^^26^ Further, Numasawa et al's^18^ report of a patient experiencing stillborn birth with SCAD as well as reports of breastfeeding women experiencing SCAD^18^ suggests that elevated levels of hormones relating to pregnancy are associated with P-SCAD.^1^^,^^12^^,^^13^^,^^30^^,^^45^ This cumulative effect theory also is supported by the findings in Tweet et al's^9^^,^^13^ studies that show a link between P-SCAD and women, mostly older, with a history of multiple births and preeclampsia.

Elevated progesterone levels during pregnancy could weaken the normal elasticity of arterial walls,^8^^,^^32^^,^^45^, ^46^, ^47^ resulting in reticular fiber fragmentation and collagen degeneration hypertrophy of the smooth muscle cells.^32^ Estrogen is believed to create a hypercoagulable condition^8^ and to lead to cystic medial necrosis.^17^ The increased serum levels of progesterone and estrogen impair the synthesis of collagen, resulting in decreased elasticity in the media layer of the artery.^17^ All these factors increase the risk of SCAD.^46^

Pregnancy causes increased cardiac output and a higher level of blood volume in the circulatory system. This, together with the effects of hormone changes during pregnancy, increase the risk of SCAD in pregnant women due to the greater stress on already weakened arteries.^8^^,^^34^^,^^46^^,^^48^ Indeed, Sharma et al^22^ note that a majority of SCAD patients with ACS were women without pregnancy history as a factor. Maas et al^35^ note there is not enough data to differentiate between P-SCAD and SCAD patients at presentation.

Because SCAD is not specifically limited to premenopausal women,^11^ the link between hormone replacement therapy and SCAD warrants further investigation,^25^^,^^35^ given that menopause and the related rapid hormone changes are also likely to contribute to SCAD.^34^ Hormone therapies may pose similar increased risks of permanently damaging the arterial walls, similar to increased hormone levels during pregnancy, thus increasing the risk of SCAD.^45^ SCAD patients on hormone replacement therapy had a higher recurrence^8^^,^^32^^,^^45^, ^46^, ^47^ of SCAD than did patients who were not. Maas et al^35^ offer a different view, noting that given the large incidence of hormone therapies in the general population, the rate of SCAD is too low to warrant a connection.^35^

### Timing of P-SCAD

The fact that P-SCAD most commonly occurs in later stages of pregnancy and in the immediate postpartum period is attributed to the rapid hormonal changes during and after birth,^35^^,^^36^ as well as the relatively long time needed (at least 6 months) for arterial tissue structure to revert to the prepregnancy condition.^48^ Anecdotal observations further indicate that breastfeeding as well as multiparity may elevate SCAD risk.^45^^,^^46^^,^^48^ Moreover, advancing maternal age may increase the risk of both MI and SCAD.^15^ However, growing evidence indicates that SCAD is more common among postmenopausal women than previously believed.^1^^,^^34^^,^^37^^,^^38^

### Treatment approaches

#### Conservative

Conservative, noninvasive SCAD treatment approach is recommended for stable patients with good blood flow.^6^^,^^8^^,^^10^^,^^17^^,^^30^^,^^32^^,^^49^^,^^50^ However, in P-SCAD patients, more aggressive treatments are often warranted, typically involving PCI. A conservative approach was found to be less effective for P-SCAD patients, often resulting in failure and the need for further treatment such as PCI and CABG procedure.

#### Revascularization or PCI

PCI is recommended only if there is poor coronary flow, persistent chest pain, or persistent ST elevation, and in case of hemodynamic instability. Although it is deemed appropriate by some authors in cases of acute SCAD,^51^ others suggest that PCI could worsen the dissection through elongation or occlusion and hinder spontaneous healing^17^^,^^52^ because the dissected arterial segments are likely to eventually heal on their own.^13^ However, it is often warranted in P-SCAD patients because a conservative approach may not yield the desired results in these cases.^13^ It is possible that P-SCAD patients tend to have more serious clinical ACS presentations, thus accentuating the need for PCI and other interventions.^12^^,^^14^ Nonetheless, it is worth noting that studies consistently show that while PCI is utilized more frequently in acute P-SCAD cases than NP-SCAD cases,^11^, ^12^, ^13^ P-SCAD patients also experience a greater rate of complications after PCI, including repeat PCI, CABG, cardiogenic shock, and maternal death.^12^ There is also increased risk for additional dissection and occlusion requiring further invasive treatments.

Havakuk et al^12^ demonstrated an unusually high incidence of CABG surgery (37%) when compared with NP-SCAD cases. P-SCAD patients are at higher risk for complications, including 44 cases (37%) requiring CABG surgery, 9 cases of cardiogenic shock (24%), 34 cases in need of mechanical support (28%), and 5 cases of maternal mortality (4%).^12^ CABG is reserved for the most serious cases or when PCI fails because the success rates for P-SCAD patients are lower than NP-SCAD patients.^6^^,^^11^ Data in the studies are consistent with the prevailing view that P-SCAD presents more acutely, requires more intervention, and is associated with lower success rates. One area to explore is the relationship of P-SCAD to other vascular abnormalities. P-SCAD cases are more acute, requiring further intervention such as PCI and CABG. Although the rates of success of those interventions in P-SCAD cases are lower than in NP-SCAD cases, what is not known is whether those lower success rates are related to the treatment itself or because it is used more often in initially critical P-SCAD cases.

#### Drug therapy

Generally, pharmacologic treatment for SCAD is an extension of conservative treatment and is typically based on medical treatment protocols similar to those adopted for ACS patients.^41^ Whereas some suggest a more nuanced approach for SCAD patients, no randomized controlled studies have been conducted.^4^ In practice, certain preferred pharmacologic approaches are being established. Because the use of statins is not indicated,^5^ nitrates, calcium-channel blockers, and ranolazine should be considered when NP-SCAD patients present with chest pain.^5^ Dual antiplatelet therapy has been suggested for up to 1 year after SCAD diagnosis, combined with an lifelong aspirin regimen,^4^^,^^5^^,^^8^ but there is no consensus on the use of dual antiplatelet therapy, given that it increases the risk of cardiac events by aggregating the dissections and causing additional bleeding.^53^ In fact, Hayes et al^4^ note the new trend is to reduce the use of dual antiplatelet therapy. Cerrato et al^53^ found that a majority of female SCAD patients treated with initial conservative management usually received dual antiplatelet therapy, and the 1-year outcome resulted in a rate of 14.6% for major cardiac events, either through MI or unplanned PCI. This occurrence rate was significantly higher than when compared with a singular antiplatelet regimen.^53^ Certain drug therapies carry greater risk and should be carefully considered for P-SCAD patients. When treating P-SCAD patients, antiplatelets should be prescribed carefully given the risks to the fetus, and similar caution is warranted before prescribing them to premenopausal women who could be at risk for menorrhagia.^4^ Finally, thrombolytic agents, often prescribed in ACS, are viewed as inappropriate for SCAD treatment because they could expand the dissection and worsen coronary spasms^8^^,^^34^^,^^54^ leading to coronary rupture.^1^^,^^5^^,^^34^^,^^41^^,^^54^

### Recurrence

SCAD recurrence is defined as a subsequent de novo SCAD in a different location within 12 months^30^ and is estimated to range from 12% to 29% in the general population.^14^^,^^30^ However, limited research on P-SCAD and NP-SCAD cohorts exists, but the available findings indicate that the risk of recurrence is relatively low.^14^^,^^23^

### Limitations

This study is based on available data reported in articles, literature reviews, and case series pertaining to the occurrence of SCAD in women, particularly P-SCAD in North America during 2006 to 2021. The study is limited by its design and data collected mostly from single case reports and smaller case series. Because this was not a meta-analysis systematic review, the results shown reflect the data identified in the reviews only and may not include all cases of P-SCAD and NP-SCAD occurring during the time frame studied. The data could reflect a selection bias for submission and publishing complicated cases. Some studies did not differentiate between P-SCAD and NP-SCAD for all predisposing conditions and associated pathologies, so that data were excluded from the analysis of those conditions to prevent bias for those conditions. Certain data from large studies using SCAD registries may result in bias due to the voluntary nature of the patient data collection at the registry itself. Finally, the small number of patients with SCAD recurrence after pregnancy or P-SCAD made it challenging to definitively identify significant differences in recurrence of SCAD between P-SCAD and NP-SCAD cases.

## Conclusions

Because SCAD primarily affects women often not presenting with typical symptoms of cardiovascular diseases during late pregnancy or postpartum, pregnant women and those planning pregnancy need to be carefully screened for any predisposing factors and physical complaints to facilitate timely diagnosis, using angiography along with blood tests to check Troponin levels. Counseling should be conducted using a multidisciplinary approach involving cardiology and obstetrics specialists. Moreover, primary care providers and emergency medicine physicians should be made aware of the main SCAD risk factors to facilitate more timely referrals when needed.

## Conflicts of Interest

The author has indicated that she has no conflicts of interest regarding the content of this article.
